# Giant cysts, no incisions: Ultrasound-guided sclerotherapy in the elderly

**DOI:** 10.1097/MD.0000000000046897

**Published:** 2025-12-26

**Authors:** Zeyang Dong, Mengyao Zhao, Jie Chen, Yuke Zhao, Xixi Sun, Bin Huang, Yaowu He

**Affiliations:** a The Second School of Clinical Medicine, Zhejiang Chinese Medical University, Hangzhou, China; b Department of Ultrasound, Zhejiang Hospital, Hangzhou, China; c Department of Ultrasound, Lijiang People’s Hospital, Lijiang, China.

**Keywords:** efficacy analysis, giant cyst, sclerotherapy, ultrasound guidance

## Abstract

Ultrasound (US)-guided sclerotherapy is effective for hepatic and renal cysts, but data on giant cysts are limited, especially in elderly patients prone to recurrence. This study assessed the efficacy of US-guided tube drainage with medical anhydrous ethanol in treating giant hepatic and renal cysts. In this dual-center retrospective study, 55 patients with giant hepatic or renal cysts underwent US-guided percutaneous tube drainage and ethanol sclerotherapy in Zhejiang Hospital and Lijiang People’s Hospital from February 2023 to February 2024. Clinical outcomes and adverse events were analyzed. Potential confounders were addressed through strict inclusion/exclusion criteria, standardized procedures, and multivariate adjustment. One year after surgery, efficacy was comparable between the ethanol flushing and flushing-retention methods. For the cyst volume reduction rate (VRR) at 3 months post-surgery, cyst type (β = 0.113, *P* < .001), treatment method (β = –0.060, *P* = .018), and maximum diameter (β = –0.009, *P* = .019) were independent influencing factors. Furthermore, age (β = 0.004, *P* = .023) and treatment method (β = –0.095, *P* = .014) were independent predictors of VRR at 1 year postoperatively. Maximum cyst diameter was the only independent factor influencing recurrence (OR = 1.35, *P* = .009). US-guided tube drainage and sclerotherapy with medical anhydrous ethanol is an effective, minimally invasive option for giant hepatic and renal cysts, offering a valuable strategy for elderly patients.

## 1. Introduction

Simple hepatic and renal cysts are among the most common benign lesions found in the liver and kidneys. They are often discovered incidentally during examinations or when symptoms arise. The primary cause of these cysts is believed to be congenital developmental abnormalities or acquired degenerative changes.^[1–3]^ In clinical practice, hepatic cysts larger than 10 cm in diameter and renal cysts larger than 8 cm are classified as giant cysts, typically seen in elderly patients.^[4]^ While simple cysts usually do not require treatment, giant liver and kidney cysts can compress surrounding organs and affect vital functions such as circulation, respiration, and digestion. This compression can lead to symptoms like chest tightness, asthma, and jaundice, which may necessitate interventional treatment.^[5–7]^

Surgical procedures, including open surgery and laparoscopic cyst debridement, are the traditional primary treatment options for liver and kidney cysts. Although these approaches are thorough and have a low recurrence rate, giant liver and kidney cysts occur most often in elderly patients who often have difficulty tolerating surgery due to poor overall health. As a result, there is a pressing need for an effective minimally invasive treatment alternative. Currently, ultrasound (US)-guided cystosclerosis is the preferred minimally invasive treatment method.^[8]^ This technique can reduce surgical trauma, shorten hospitalization time, and lessen the overall burden of medical care, making it particularly effective and safe for elderly patients.

Aspiration sclerotherapy for giant cysts faces several challenges, including high difficulty in the procedure, low absorption rates, numerous adverse reactions, and a high recurrence rate. The specific clinical efficacy of various sclerotherapy methods for treating giant cysts remains unclear. In this study, we aim to explore the clinical value of US-guided aspiration sclerotherapy for the treatment of giant hepatic and renal cysts, as well as to evaluate its effectiveness.

## 2. Materials and methods

### 2.1. Participants

A total of fifty-five patients with large liver and kidney cysts underwent US-guided percutaneous tube drainage and medical anhydrous ethanol sclerotherapy at Zhejiang hospital and Lijiang People’s Hospital from February 2022 to February 2024, all patients are treated by the same doctor. Patients were selected according to the following criteria: simple hepatic cysts or large polycystic liver cysts with an US-measured maximum section diameter of ≥10 cm; simple renal cysts or large polycystic kidney cysts with an US-measured maximum section diameter of ≥8 cm; and Patients experiencing clinical symptoms due to cyst compression. The exclusion criteria were as follows: hepatic cysts connected to intrahepatic bile ducts; renal cysts originating from the renal pelvic; cysts with malignant lesions that could not be ruled out; allergies to sclerosing agents; severe hepatic and renal dysfunction; coagulation disorders in patients on anticoagulant therapy; absence of a safe puncture route; patients unable to tolerate the treatment; and patients with incomplete clinical data.

Only patients without severe comorbidities (e.g., cardiovascular disease, advanced chronic kidney disease, decompensated liver cirrhosis) were included. Preoperative liver and kidney function parameters showed no statistically significant differences between groups. To minimize operator-related variability, all procedures were performed by the same senior chief physician with extensive experience in cyst sclerotherapy.

The study examined 2 types of cysts: liver cysts and renal cysts. There were 33 cases in the liver cyst group and 22 cases in the renal cyst group. These groups were further divided into a flushing group and a flushing retention group based on the sclerotherapy method used. This retrospective study followed the guidelines outlined in the “Chinese Expert Consensus on Sclerotherapy for cysts of multiple organs (2021 Edition),”^[8]^ which had received approval from Zhejiang Hospital’s Ethics Committee (No. ZJHIRB-2025-055K). Given its retrospective design, the use of anonymized data, the requirement for written informed consent was waived in accordance with the Declaration of Helsinki and the regulations of the Ethics Committee.

### 2.2. Equipment and materials

The GE-LOGIQ E11 US scanner, manufactured by General Electric Healthcare Co. in Wauwatosa, Wisconsin, USA, was utilized for patient examinations. A probe frequency of 3.5 to 5.0 MHz was used during the procedure. Hardner, a dehydrated alcohol injection with a concentration of C_2_H_6_O ≥ 96.8% (g/g), was supplied by SINOPHARM Co. in Anhui, China. The procedure employed a 6F Straight Drainage Catheter provided by DIALL Medical Technology. Co. in Zhengzhou, China. Lidocaine hydrochloride for injection was sourced from KELUNPHARM. Co. in Hunan, China.

### 2.3. Procedures

**Figure 1.**
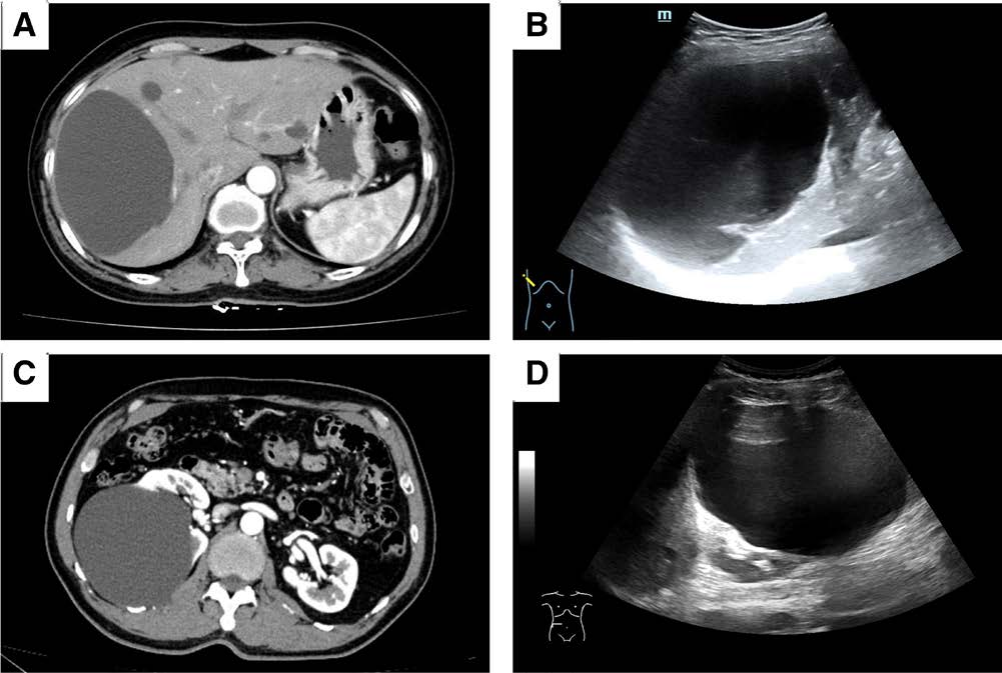
Preoperative CECT and US to evaluate the size and location of hepatic and renal cyst. (A) Characterization of giant hepatic cysts in CECT. (B) Ultrasonographic image of a giant hepatic cyst. (C) Characterization of giant renal cyst in preoperative CECT and exclusion of other lesions. (D) Giant renal cyst in US sonography. CECT = contrast-enhanced computed tomography, US = ultrasound.

contrast-enhanced computed tomography, US, contrast-enhanced US and magnetic resonance imaging were conducted to assess the size and location of the lesions and to rule out malignant lesions and specific cysts (Fig. 1). Additionally, preoperative tests were performed to evaluate coagulation function, liver function, kidney function, and other relevant indicators.

**Figure 2.**
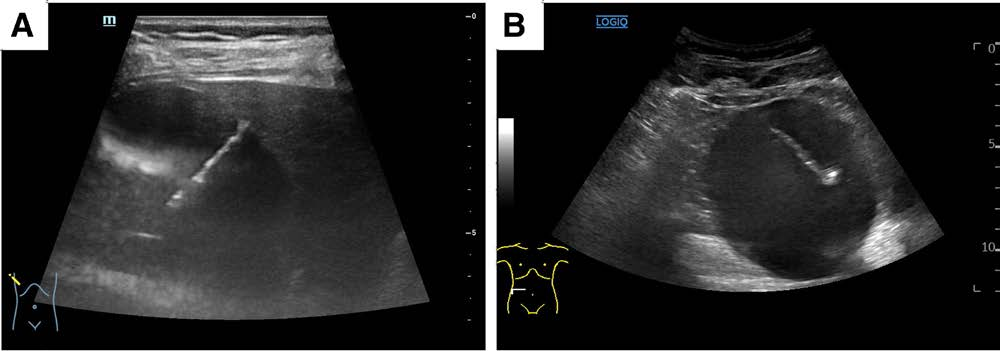
US-guided tube drainage for giant hepatic and renal cyst. (A) A drain is placed into the cyst via puncture of normal liver tissue. (B) US-guided placement of drainage tubes in renal cyst. US = ultrasound.

The patients were placed in a supine position or lateral position. Select the appropriate puncture point and determine the needle path. Perform routine disinfection and arrange towels as needed. Administer local infiltration anesthesia to the liver and renal. Instruct the patient to hold their breath. Utilize US guidance to avoid any blood vessels, nerves, or important tissue structures while inserting the needle (Fig. 2). Use a disposable 6F drainage tube to access the cyst and extract fluid. If the patient is weak or if volume of the cystic is excessive, consider placing the tube for intermittent drainage initially. Take the cystic fluid for protein characterization testing and send it for pathological examination. If the protein characterization test results are atypical or if the cyst is particularly large, perform intracapsular ultrasonography to rule out malignant lesions or specific types of cysts.

**Figure 3.**
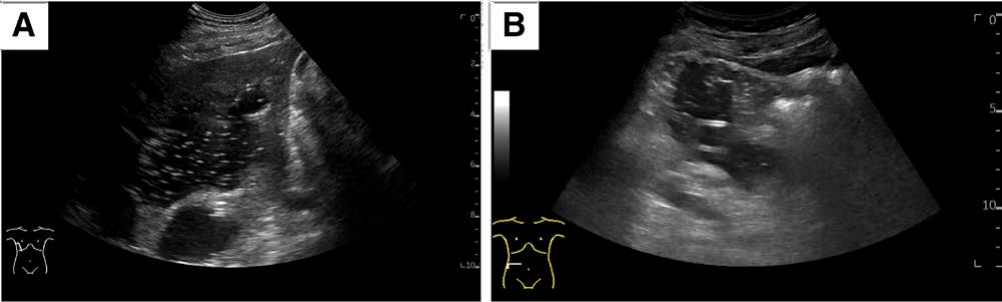
US-guided sclerotherapy for giant hepatic and renal cyst. (A) Sclerotherapy with an injection of medical anhydrous ethanol after evacuation of hepatic cystic fluid. (B) Sclerotherapy using the injection of medical anhydrous ethanol following the drainage of renal cystic fluid. US = ultrasound.

If the predicted volume of cystic fluid exceeded 3000 mL, the patient underwent intermittent drainage for several days. During this period, the cystic fluid was pumped out through a drainage tube. Once the capsule wall collapsed and retracted, a 1% lidocaine solution was used to flush the capsule cavity. This lidocaine was retained for one minute before being withdrawn to minimize discomfort for the patient. Following the flushing, medical anhydrous ethanol sclerosant was injected into the capsule cavity. The volume of sclerosant used was approximately one-fourth of the total volume of the suctioned fluid, with a maximum limit of 120 mL per session (Fig. 3). Patients were instructed to change their positions to ensure that the sclerosant made full contact with the capsule wall. The sclerosant was retained in the capsule for 5 to 10 minutes before being withdrawn. In the flushing group, the drain was removed after the sclerosing has been withdrawn. Additionally, 5 mL of medical anhydrous ethanol was injected into the capsule once more after the sclerosing agent has been removed, at which point the drainage tube was taken out.

### 2.4. Observation indicators

#### 2.4.1. Efficacy evaluation

**Figure 4.**
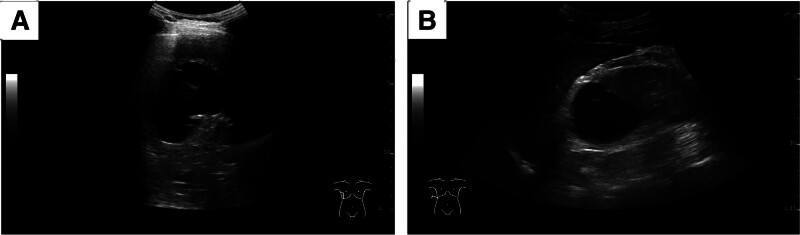
Follow-up US conducted one year after surgery indicated a significant reduction in the cyst present in the liver and kidney. (A) Liver US reveals a significant decrease in the size of a previously giant liver cyst following US-guided drainage and sclerotherapy. (B) Considerable reduction of a large renal cyst on US one year after US-guided drainage and sclerotherapy. US = ultrasound.

The efficacy of cystosclerosis was assessed after surgery by evaluating the improvement of clinical symptoms alongside the reduction rate of cyst volume. Patients were instructed to undergo US examinations to monitor the size of the cysts at 1, 3, 6, and 12 months following surgery (Fig. 4). The volume reduction ratio (VRR) is calculated using the formula: VRR = (pretreatment volume − cyst volume at review)/ pretreatment volume × 100%. Cyst volume (in milliliters) is determined using the formula on diameter measurement with US: *V* (mL) = *ab*c × π/6 (*V* = volume; *a* = the largest diameter; *b* and *c* = the other 2 perpendicular diameters). The changes in cyst volume before and after treatment were compared using postoperative imaging examinations at 3 months and 12 months following surgery. The main evaluation criteria were as follows: Cure: VRR > 90% one year postoperatively, with disappearance of clinical symptoms; Effective: VRR between 51% and 90% one year after surgery, with clinical symptoms relieved or disappeared; Ineffective: VRR ≤ 50% one year after surgery or enlarged from the previous volume, with no improvement of clinical symptoms; and Recurrence: after the cyst shrinks or clinical symptoms improve after surgery, but a follow-up shows an increase in the cyst volume of more than 50%, or if clinical symptoms worsen again, while excluding the possibilities of intracapsular hemorrhage, infection, and new lesions.^[9,10]^

### 2.5. Hematological indicators

Preoperative and 3-month postoperative hematological parameters for patients with hepatic cysts included alanine aminotransferase, aspartate aminotransferase, and total bilirubin. For patients with renal cysts, parameters included creatinine (Cr) and blood urea nitrogen (BUN).

### 2.6. Complications

During the operation, the patient’s vital signs and level of consciousness were closely monitored for any signs of drunkenness reaction, such as agitation, excessive sweating, or flushing. After the surgery, patients were assessed for pain using the numerical rating scale (NRS) and were observed for their overall condition, potential adverse reactions, and complications. The patient’s body temperature was measured postoperatively to check for any signs of fever. An US examination was repeated one day after surgery.

### 2.7. Statistical analysis

All statistical analyses were done using IBM SPSS Statistical 26.0 (IBM Corp., Armonk). Continuous variables were assessed for normality by the Shapiro–Wilk test, with independent samples *t*-tests for normal distributions and Mann–Whitney *U*-tests for non-normal distributions. Normal count data were expressed as ($\bar{\chi }\pm s$), skewed count data were expressed as M (P25, P75), and categorical data were expressed as a rate (%). Categorical variables were analyzed for differences between groups using the Fisher exact test. Between-group comparisons of recurrence were performed using the log-rank test, and logistic regression was used to analyze factors associated with recurrence. For the continuous quantitative variable of postoperative cyst volume resorption rate, linear regression was used to analyze the influencing factors associated with postoperative cyst resorption rate.

Differences between variables were considered statistically significant at a *P *< .05, and all comparisons were two-sided tests, while the advantage ratio (OR), regression coefficient (β), and their 95% confidence intervals were reported.

### 2.8. post hoc analysis

In this study, postoperative cyst VRR was used as the primary outcome indicator to estimate the sample size. When the sample size was 33 and 22 for the liver and kidney cyst groups, respectively, the test efficacy was 99.97% (significance level (α) was set at 0.05) based on the effect value and the mean and standard deviation of the samples, which was calculated using G*Power (version 3.1.9.7). Therefore, the sample size in this study can be considered sufficient for evaluating the accuracy and stability of the results.

## 3. Results

### 3.1. Baseline characteristics

A total of 55 patients including 22 patients with renal cysts and 33 patients with hepatic cysts were included in this study. There were 26 males and 29 females, the mean age was (66.18 ± 8.48) years in the hepatic cyst group and (64.00 ± 12.11) years in the renal cyst group. The maximal diameter of hepatic cysts was 10.3 (8.6, 11.0) cm, whereas that of renal cysts was 8.4 (8.2, 10.0) cm, and the maximal diameter and predicted volume of cystic fluid of the hepatic cysts were greater than those of renal cysts, and the difference between the groups was statistically significant. However, given the difference in organ size, the ratio of the maximum diameter of the cyst to the maximum diameter of the organ was calculated to be 58.4% (56.7, 59.5) for hepatic cysts and 58.9% (57.9, 63.0) for renal cysts, and the difference between the groups was not statistically significant (*P* = .115). There was no statistically significant difference in the preoperative baseline data between the 2 groups (Table 1).

**Table 1:** Comparison of the baseline data between the hepatic cysts group and renal cysts group.

Variables	Hepatic cysts group	Renal cysts group	*t*(χ^2^)	*P*
Gender (%)				
Male	13 (39.4)	13 (59.1)	2.055	.152
Female	20 (60.6)	9 (40.9)		
Age (yr)	66.18 ± 8.48	64.00 ± 12.11	0.787	.435
Treatment methods (%)				
Flushing	24 (72.7)	15 (68.2)	0.132	.716
Flushing retention	9 (27.3)	7 (31.8)		
Maximum diameter of the cyst (cm)	10.3 (8.6, 11.0)	8.4 (8.2, 10.0)	−4.637	<.001
Maximum diameter ratio of cyst to the viscera (%)	58.4 (56.7, 59.5)	58.9 (57.9, 63.0)	−1.574	.115
Predicted cystic fluid volume (mL)	384.3 (228.1, 496.6)	173.3 (139.0, 286.1)	−3.694	<.001

The value of the maximum diameter of the cyst was measured by US.

US = ultrasound.

### 3.2. Hematology results

Among patients with same type cysts, the differences in preoperative and postoperative findings of alanine aminotransferase, azelaic acid transaminase (aspartate aminotransferase), glutamic acid aminotransferase (total bilirubin), glutamic oxalate aminotransferase (Cr), and BUN were not statistically significant between patients treated with intracapsular anhydrous ethanol rinsing sclerosis and the rinsing-retention method. However, in some of the patients treated with the flushing method in the group of giant renal cysts, Cr and BUN levels decreased after the operation, and the differences in the indicators were statistically significant when compared with the preoperative period. There was no statistically significant difference in other indices compared with preoperative period (Table 2).

**Table 2:** Comparison of preoperative and postoperative hematologic indices in the hepatic cysts group and renal cysts group.

Variables		Flushing group	Flushing retention group	*t*	*P*
Hepatic cysts					
ALT (U/L)	Pre-op	30.96 ± 15.32	35.78 ± 11.50	−0.85	.399
	Post-op	29.67 ± 13.20	33.78 ± 10.27	−0.84	.407
AST (U/L)	Pre-op	34.92 ± 11.03	43.00 ± 9.81	−1.93	.063
	Post-op	36.08 ± 14.07	41.22 ± 13.82	−0.94	.355
TBil (μmol/L)	Pre-op	16.78 ± 5.82	18.46 ± 5.98	−0.73	.471
	Post-op	16.25 ± 4.89	16.66 ± 4.57	−0.22	.828
Renal cysts					
Cr (μmol/L)	Pre-op	75.80 ± 30.02	81.86 ± 34.64	−0.42	.679
	Post-op	66.07 ± 21.76*	75.57 ± 23.99	−0.40	.366
BUN (mmol/L)	Pre-op	6.61 ± 2.27	6.63 ± 3.02	−0.02	.988
	Post-op	5.85 ± 2.01*	5.98 ± 1.87	−0.14	.891

ALT = alanine aminotransferase, AST = aspartate aminotransferase, BUN = blood urea nitrogen, Cr = creatinine, Post-op = postoperative, Pre-op = preoperative, TBil = total bilirubin.

* Refers to a *P*-value of < .05 compared to preoperative.

### 3.3. Postoperative adverse reaction

Mild adverse reactions were observed in both groups following surgery, with intraoperative pain, fever, and drunkenness reactions being the primary reactions. According to the NRS score, the renal cyst group had a score of 2 (1, 2), while the hepatic cyst group scored 1 (1, 2), and this difference was not statistically significant (*P* = .221). Postoperative fever occurred in 3 patients from the hepatic cyst group and 1 from the renal cyst group, with this difference also being statistically insignificant (*P* = .916). 2 cases of giant liver cysts in the hepatic cyst group experienced drunkenness reactions, manifested as flushing and increased speech, but both resolved spontaneously without intervention after surgery. Fortunately, there were no severe of fatal complications reported in any of the cases.

### 3.4. Follow-up VRR results

A total of 55 lesions were treated with US-guided catheter drainage and sclerotherapy. The 3-month postoperative VRR in the hepatic and renal cyst groups were (79.79 ± 10.49) % and (92.77 ± 5.94) %, respectively; and the one-year postoperative shrinkage rates were 92.24% (87.22, 93.93) and 98.36% (96.53, 99.65), respectively, with statistically significant differences (*P *< .001) (Table 3).

**Table 3:** Comparison of the sclerotherapy efficacy between the hepatic cysts group and renal cysts group.

Group	3-mo VRR (%)	12-mo VRR (%)
Hepatic cysts	79.79 ± 10.49	92.24 (87.22, 93.93)
Renal cysts	92.77 ± 5.94	98.36 (96.53, 99.65)
*t (Z*)	−5.84	−3.46
*P*	<.001	<.001

VRR = volume reduction ratio.

In the hepatic cyst group, 24 cases were treated with sclerotherapy by the flushing method and 9 cases were treated by the flushing-preserved method, and the VRR at 3 months after surgery was (79.86 ± 10.40) % versus (79.61 ± 11.37) %, respectively; and the VRR at one year after surgery was (91.68 ± 4.01) % versus (84.93 ± 15.04) %, respectively, and the difference between the 2 methods was not statistically significant (*P* = .141). In the giant renal cyst group, 15 cases were treated with the flushing method and 7 cases with the flushing-retention method, the VRR was (94.59 ± 4.34) % versus (88.87 ± 7.31) % at 3 months postoperatively and 99.07% (96.83, 100.00) versus 97.83% (82.25, 98.20) at one year postoperatively, respectively. The difference between these 2 methods was not statistically significant (*P *= .060) (Table 4).

**Table 4:** Comparison of the sclerotherapy methods in the hepatic cysts group and renal cysts group.

Type of cysts	Treatment methods	3-mo VRR (%)	12-mo VRR (%)
Hepatic cysts	Flushing	79.86 ± 10.40	91.68 ± 4.01
	Flushing retention	79.61 ± 11.37	84.93 ± 15.04
	*t (Z*)	0.06	1.32
	*P*	.952	.141
Renal cysts	Flushing	94.59 ± 4.34	99.07 (96.83, 100.00)
	Flushing retention	88.87 ± 7.31	97.83 (82.25, 98.20)
	*t (Z*)	1.919	−1.879
	*P*	.091	.060

VRR = volume reduction ratio.

### 3.5. Analysis of influencing factors postoperative VRR

We analyzed the factors affecting the VRR at 3 months and 12 months postoperatively after sclerotherapy with medical anhydrous ethanol for cysts, and linear regression analysis was performed because the data were continuous and quantitative, and the residuals conformed to the normality test.

Univariate linear regression analysis of the VRR at 3 months postoperatively revealed that cyst type (β = 0.130, *P* < .001), treatment methods (β = −0.069, *P* = .032), maximum cyst diameter (β = −0.016, *P* < .001), and predicted cyst volume (β = −0.001, *P*  = .002) were significantly associated with the 3-month postoperative VRR. In the multivariate linear regression model, cyst type (β = 0.113, *P* < .001), treatment methods (β = −0.060, *P* = .018), and maximum cyst diameter (β = −0.009, *P* = .019) were found to be the independent influences (Table 5). Patients with renal cysts had an 11.3% higher VRR at 3 months postoperatively compared to those with hepatic cysts. The use of the flushing indwelling method was associated with a 6.0% reduction in VRR compared to flushing sclerotherapy alone. Additionally, each 1-cm increase in maximum cyst diameter was associated with a 0.9% decrease in VRR at 3 months.

**Table 5:** Linear regression table for the rate of cyst resorption at 3 mo postoperatively.

Variables	Univariate regression		Multiple factor regression	
	*P*	β (95% CI)	*P*	β (95% CI)
Gender				
Female	–	0.000 (Reference)	–	–
Male	.948	−0.002 (−0.061–0.057)	–	–
Age	.84	−0.000 (−0.003–0.003)	–	–
Type				
Hepatic cyst	–	0.000 (Reference)	–	0.000 (Reference)
Renal cyst	<.001	0.130 (0.081–0.178)	<.001	0.113 (0.066–0.159)
Treatment methods				
Flushing	–	0.000 (Reference)	–	0.000 (Reference)
Flushing retention	.032	−0.069 (−0.131 to −0.008)	.018	−0.060 (−0.108 to −0.012)
Maximum diameter of the cyst	<.001	−0.016 (−0.024 to −0.009)	.019	−0.009 (−0.016 to −0.002)
Maximum diameter ratio of cyst to the viscera	.057	−0.316 (−0.635–0.003)	–	–
Predicted cystic fluid volume	.002	−0.001 (−0.990 to −0.001)	.691	–
Medical absolute ethanol dosage	.163	−0.001 (−0.002–0.000)	–	–

CI = confidence interval.

Univariate linear regression analysis of VRR at 12 months postoperatively revealed that age (β = 0.004, *P *= .023) and treatment methods (β = −0.099, *P* = .014) were significantly associated with VRR at 12 months postoperatively. In the multivariate linear regression model, both age (β = 0.004, *P* = .023) and treatment methods (β = −0.095, *P* = .014) remained independent predictors (Table 6). Compared with the rinse-and-harden method, the rinse-and-retain approach was associated with a 9.5% lower VRR at 12 months, while each additional year of age was linked to a 0.4% increase in VRR.

**Table 6:** Linear regression table for the rate of cyst resorption at 12 mo postoperatively.

Variables	Univariate regression		Multiple factor regression	
	*P*	β (95% CI)	*P*	β (95% CI)
Gender				
Female	–	0.000 (Reference)	–	–
Male	.078	−0.065 (−0.136–0.006)	.177	–
Age	.023	0.004 (0.001–0.008)	.023	0.004 (0.001–0.007)
Type				
Hepatic cyst	–	0.000 (Reference)	–	–
Renal cyst	.494	0.026 (−0.048–0.101)	–	–
Treatment methods				
Flushing	–	0.000 (Reference)	–	0.000 (Reference)
Flushing retention	.014	−0.099 (−0.175 to −0.023)	.014	−0.095 (−0.168 to −0.022)
Maximum diameter of the cyst	.140	−0.008 (−0.019–0.003)	–	–
Diameter ratio	.200	−0.269 (−0.676–0.138)	–	–
Predicted cystic fluid volume	.074	−0.000 (−0.000–0.000)	.452	–
Medical absolute ethanol dosage	.066	−0.001 (−0.002–0.000)	.282	–

CI = confidence interval.

### 3.6. Postoperative recurrence

Postoperative follow-up revealed that 7 cases experienced a recurrence, defined as a cyst volume increase of more than 50% within one year after surgery. This included 5 case in the hepatic cyst group and 2 cases in the renal cyst group; however, the difference was not statistically significant (*P* = .804). In the hepatic cyst group, there were 2 cases where the maximum diameter of the cysts exceeded 20 cm. Despite the VRR exceeding 50% one-year post-surgery, the maximum diameters of the remaining cysts were still >10 cm. Patients were advised to undergo sclerotherapy again if clinical symptoms worsened.

We conducted logistic regression analysis to evaluate factors associated with cyst recurrence following anhydrous ethanol sclerotherapy. Univariate logistic regression revealed that both the maximum cyst diameter (OR = 1.35, *P* = .009) and the predicted cyst volume (OR = 1.01, *P* = .045) were significantly associated with recurrence. Subsequent multivariate logistic regression identified the maximum cyst diameter (OR = 1.35, *P* = .009) as an independent predictor of postoperative recurrence (Table 7).

**Table 7:** Logistic regression table for recurrence after cystosclerotic surgery.

Variables	Univariate regression		Multiple factor regression	
	*P*	OR (95% CI)	*P*	OR (95% CI)
Gender				
Female	–	1.00 (Reference)	–	–
Male	.187	3.214 (0.566–18.243)	–	–
Age	.446	1.031 (0.953–1.116)	–	–
Type				
Hepatic cyst	–	1.000 (Reference)	–	–
Renal cyst	.513	0.560 (0.099–3.182)	–	–
Treatment methods				
Flushing	–	1.000 (Reference)	–	–
Flushing retention	.974	0.971 (0.168–5.612)	–	–
Maximum diameter of the cyst	.009	1.350 (1.078–1.690)	.009	1.350 (1.078–1.690)
Predicted cystic fluid volume	.045	1.001 (1.001–1.002)	.528	–
Medical absolute ethanol dosage	.689	0.994 (0.967–1.022)	–	–

CI = confidence interval, OR = odds ratio.

## 4. Discussion

The increasing use of imaging tests has led to a rise in the detection of liver and kidney cysts, particularly among elderly patients. Giant hepatic and renal cysts can cause many symptoms, and the complexity of underlying condition in elderly often makes them unable to tolerate general anesthesia for surgical procedures. As a result, finding safe treatment options for giant hepatic and renal cysts in elderly remains a significant clinical challenge.

With the development of US interventional technology, US-guided tube drainage and sclerotherapy have emerged as minimally invasive and effective treatment options.^[11,12]^ Guanglun^[13]^ compared the efficacy of polyguillin sclerotherapy with laparoscopic decortication for treating simple renal cysts. The study found that the operative time and treatment burden for patients undergoing sclerotherapy were significantly lower, leading to a recommendation for sclerotherapy as the first choice of treatment. Furumaya^[10]^ conducted a systematic review of studies comparing percutaneous perforation sclerotherapy, laparoscopic cyst decortication, and open surgery for the treatment of simple hepatic cysts, concluding that sclerotherapy demonstrated excellent efficacy and should be recommended as the first choice of treatment, with laparoscopic cyst decortication serving as an alternative for recurrent cysts after sclerotherapy.

The choice of sclerosing agent plays a crucial role in the treatment efficacy and safety of cystic lesions. Both polycinnamic alcohol and anhydrous ethanol exert their effects by damaging the endothelial lining of the cyst wall. Previous studies have demonstrated that anhydrous ethanol is an effective option for treating giant liver cysts, with comparable efficacy to polycinnamic alcohol but a higher incidence of adverse events.^[14,15]^ Previous literature has reported that anhydrous ethanol sclerotherapy may cause significant pain, which may interrupt the procedure and limit its clinical use. Hao^[16]^ discovered that a modified single anhydrous ethanol sclerotherapy technique for treating simple renal cysts could save operative time, reduce patient discomfort, and achieve excellent clinical outcomes. In this study, 1% lidocaine was used to flush the cystic cavity before the administration of medical anhydrous ethanol sclerosis. The modified technique successfully reduced intraoperative pain, and the difference in NRS scores between the hepatic and renal cyst groups was not statistically significant.

The adverse effects of anhydrous ethanol are closely related to the administered dose.^[15]^ It is generally recommended that the maximum volume for a single sclerotherapy session should not exceed 120 mL. However, in large cysts with substantial fluid content, the injected ethanol often represents less than one-fourth of the cyst volume, which may result in suboptimal treatment outcomes. In this study, sclerotherapy was performed by injecting medical-grade anhydrous ethanol into the cyst cavity via a disposable 6F drainage catheter. Patients were instructed to change their position repeatedly to ensure more uniform contact of the sclerosing agent with the cyst wall. For very large cysts, this procedure was repeated 2 to 3 times.

After drainage, large cysts often exhibit wall collapse or wrinkling, which can limit ethanol distribution. Positional adjustments help overcome this issue, enhancing the agent’s efficacy while minimizing the required dose and reducing the risk of adverse effects. In our cohort, only 3 cases of ethanol-related intoxication were observed, all occurring in patients who received the full 120 mL dose. Postoperative interviews indicated that these patients denied habitual alcohol consumption but reported a predisposition to alcohol intolerance, such as becoming easily intoxicated after minimal alcohol intake. This suggests a reduced capacity to metabolize ethanol, which may underlie the intoxication-like reactions observed after sclerotherapy.^[17]^

In anhydrous ethanol sclerotherapy for cyst treatment, the chosen method can influence therapeutic efficacy. Several studies have reported no statistically significant differences in volume reduction or symptomatic improvement at 3 months post-procedure, regardless of variations in intracystic retention times of ethanol.^[18–20]^ Although limited data exist on the efficacy of sclerotherapy for hepatic and renal cysts, the present study specifically compared 2 techniques for treating giant hepatic and renal cysts. Our results showed no statistically significant differences in reduction rates at both 3 months and one year postoperatively. Nevertheless, given the small sample size, further research is warranted to clarify the relationship between treatment efficacy and the volume of anhydrous ethanol retained within the cyst.

To further assess the efficacy of sclerotherapy, we performed linear correlation and regression analyses of cyst VRRs (VRR) at 3 and 12 months postoperatively. A significant positive correlation was observed between the 3-month and 12-month VRRs (*R* = 0.403, *P* = .002), suggesting that early cyst shrinkage may serve as a predictor of long-term treatment outcomes.

Multiple linear regression analysis identified cyst type (β = 0.113, *P* < .001), sclerotherapy modality (β = –0.060, *P* = .018), and maximum cyst diameter (β = –0.009, *P* = .019) as independent predictors of 3-month VRR. These findings indicate that larger cysts tend to show poorer short-term responses. Additionally, the flush-only sclerotherapy approach demonstrated superior short-term efficacy compared with the flush-retention technique. Renal cysts also exhibited better short-term responses than hepatic cysts, likely due to histological and anatomical differences. Specifically, renal cysts have thinner walls, less fibrosis, and lower metabolic activity, facilitating more complete penetration of anhydrous ethanol throughout the cyst wall and thereby enhancing therapeutic effectiveness.^[21,22]^

In contrast, age (β = 0.004, *P* = .023) and sclerotherapy modality (β = –0.095, *P* = .014) were identified as independent predictors of 12-month postoperative VRR. The flush-only approach continued to demonstrate superior long-term efficacy compared with the flush-retention method. Interestingly, older patients exhibited better long-term outcomes, which may be related to reduced epithelial activity of the cyst lining in the elderly, facilitating prolonged action of the sclerosing agent, as well as the tendency toward chronic inflammation and fibrosis, promoting fibrous scar formation following capsule wall closure.^[23–25]^ This finding suggests that US-guided minimally invasive treatment of giant hepatic and renal cysts may achieve particularly favorable outcomes in elderly patients, warranting further investigation.

Multivariate logistic regression analysis identified maximal cyst diameter (OR = 1.35, *P* = .009) as an independent predictor of recurrence after treatment. This indicates that larger cysts are more prone to recurrence despite initial treatment success. Incomplete eradication of the cyst by a single ethanol sclerotherapy session may leave residual epithelial lining capable of fluid secretion, contributing to recurrence. Larger cysts also present technical challenges in achieving uniform ethanol distribution and effective contact with the cyst wall, both critical for successful sclerosis.^[26,27]^ Clinically, these findings highlight the importance of preoperative assessment of cyst size. For particularly large cysts, strategies such as instructing patients to change position post-injection to enhance cyst wall contact, or employing combinations of multiple sclerosing agents, may improve outcomes. Patients with recurrent giant hepatic or renal cysts should be counseled on the likelihood of recurrence and the potential need for secondary sclerotherapy to optimize long-term results. In frail patients with poor baseline status, treatment may be deferred, with regular follow-up recommended to monitor cyst progression.

In addition to the demonstrated clinical efficacy, the broader applicability of US-guided ethanol sclerotherapy warrants consideration. The technique is relatively inexpensive and requires a shorter learning curve compared with other interventional procedures, which supports its potential adoption in resource-limited settings. Nevertheless, the scarcity of trained interventional radiologists in rural or underserved regions may limit accessibility, highlighting the need for targeted training programs for local healthcare providers. For hepatic cysts in particular, the risk of biliary communication remains a critical concern, as inadvertent ethanol entry into the biliary system can lead to serious complications. Thorough pre-procedural imaging to delineate cyst–bile duct anatomy, cautious ethanol dosing, and immediate cessation of injection if bile is aspirated are essential preventive strategies. Addressing these practical and safety considerations could facilitate wider and safer use of this minimally invasive treatment across diverse clinical settings.

This study has several limitations that should be acknowledged. First, it was a retrospective analysis, which may introduce selection bias. Second, the relatively small sample size reflects the low prevalence of patients with giant hepatic and renal cysts. Consequently, multicenter studies with larger cohorts are warranted to validate these findings. Although the hepatic cysts in this study had greater maximum diameters than renal cysts, this difference reflects inherent anatomical variations between the liver and kidney. When adjusted for organ size, the ratio of cyst diameter to the corresponding organ’s maximum diameter did not differ significantly between groups, indicating balanced baseline characteristics. Additionally, cysts treated with the intracystic ethanol retention method were generally larger in volume than those managed with the flushing technique. Given the retrospective design, the choice of treatment modality may have been influenced by clinical judgment, introducing potential selection bias. Therefore, further prospective studies are needed to directly compare the clinical efficacy and safety of different sclerotherapy approaches, particularly for giant hepatic and renal cysts.

## 5. Conclusions

In conclusion, US-guided tube drainage and sclerotherapy is an effective treatment for giant liver and kidney cysts, especially for elderly patients who cannot tolerate surgery. In this study, we demonstrated that anhydrous ethanol was more efficacious in the treatment of giant renal cysts. In addition, there was no statistically significant difference in efficacy between the anhydrous ethanol flushing method and the flushing-retention method. In conclusion, US-guided tube drainage and sclerotherapy show strong efficacy in the treatment of giant hepatic and renal cysts, providing a new strategy for the treatment of elderly patients with giant cysts.

## Author contributions

**Conceptualization:** Zeyang Dong, Bin Huang.

**Data curation:** Zeyang Dong, Mengyao Zhao, Jie Chen, Yuke Zhao.

**Formal analysis:** Zeyang Dong, Mengyao Zhao, Yuke Zhao.

**Funding acquisition:** Yaowu He.

**Investigation:** Zeyang Dong, Jie Chen, Bin Huang.

**Methodology:** Zeyang Dong, Mengyao Zhao, Jie Chen, Yuke Zhao.

**Project administration:** Xixi Sun, Bin Huang.

**Resources:** Xixi Sun, Yaowu He.

**Supervision:** Zeyang Dong, Xixi Sun, Bin Huang, Yaowu He.

**Validation:** Zeyang Dong, Mengyao Zhao, Yaowu He.

**Visualization:** Zeyang Dong, Mengyao Zhao.

**Writing – original draft:** Zeyang Dong, Mengyao Zhao, Bin Huang.

**Writing – review & editing:** Zeyang Dong, Mengyao Zhao, Jie Chen, Yuke Zhao, Xixi Sun, Bin Huang, Yaowu He.
